# Isolation, production, purification and characterization of an organic-solvent-thermostable alkalophilic cellulase from *Bacillus vallismortis* RG-07

**DOI:** 10.1186/s12896-015-0129-9

**Published:** 2015-03-19

**Authors:** Rajeeva Gaur, Soni Tiwari

**Affiliations:** Department of Microbiology (Centre of Excellence), Dr. Ram Manohar Lohia Avadh University, Faizabad, 224001 Uttar Pradesh India

**Keywords:** *Bacillus vallismortis*, 16S rDNA, Solvent tolerant, Heavy metal, Thermotolerant

## Abstract

**Background:**

The rising concerns about the scarcity of fossil fuels, the emission of green house gasses and air pollution by incomplete combustion of fossil fuel have also resulted in an increasing focus on the use of cellulases to perform enzymatic hydrolysis of the lignocellulosic materials for the generation of bioethanol. The aim of this study was to isolate a potential thermo-solvent tolerant cellulase producing bacterium from natural resources, and then applied for purification and characterization. The purified enzyme was to be accessible for the bioethanol production as well as industrial exploitation (discuss in our next study).

**Results:**

It is the first instance when thermo-solvent tolerant cellulase producing bacterium was isolated from soil sample. The culture was identified as *Bacillus vallismortis* RG-07 by 16S rDNA sequence analysis. *Bacillus vallismortis* RG-07 reported maximum cellulase production from sugarcane baggase (4105 U ml^−1^) used as agro-waste carbon source. The cellulase enzyme produced by the *Bacillus* sp. was purified by (NH_4_)_2_SO_4_ precipitation, ion exchange and gel filtration chromatography, with overall recovery of 28.8%. The molecular weight of purified cellulase was 80 kDa as revealed by SDS-PAGE and activity gel analysis. The optimum temperature and pH for enzyme activity was determined as 65°C and 7.0 and it retained 95 and 75% of activity even at 95°C, and 9.0 respectively. The enzyme activity was enhanced in the presence of organic solvents (30%) n-dodecane, iso-octane, n-decane, xylene, toluene, n-haxane, n-butanol, and cyclohexane, after prolonged incubation (7 days). The enzyme activity was also stimulated by Ca^2+^, mercaptoethanol, Tween-60, and Sodium hypochloride whereas strongly inhibited by Hg. Kinetic analysis of purified enzyme showed the K_m_ and V_max_ to be 1.923 mg ml^−1^ and 769.230 μg ml^−1^ min^−1^, respectively.

**Conclusion:**

The unique property of solvent-thermostable-alkalophilic, nature proves the potential candidature of this isolate for current mainstream biomass conversion into fuel and other industrial process.

**Electronic supplementary material:**

The online version of this article (doi:10.1186/s12896-015-0129-9) contains supplementary material, which is available to authorized users.

## Background

In present time, Cellulases have attracted much attention because of their application in various industrials processes, including food, textiles, laundry, pulp and paper as well as in agriculture [1]. Cellulases contribute to 8% of the worldwide industrial enzyme load and the demand is expected to increase by 100% within 2014 [2]. The increasing concern about the shortage of remnant energy, the release of green house gasses by incomplete burning of fossil fuel which create air pollution have resulted in rising center on the use of cellulases to carry out enzymatic hydrolysis of the lignocellulosic waste materials for the production of bioethanol [3,4]. Cellulases have a group of three enzymes namely endo-1,4-β-glucnase (Endogluconase), exo-1,4-β-glucnase (Exoglucanases) and β-glucosidase that synergistically hydrolyzed cellulose into soluble sugars and glucose [5]. Endogluconases attack the cellulose crystalline structure at random places, breaking the linear chains of glucose molecules to produce shorter chains. Each break generates two new chain ends. Exoglucanases act to these exposed ends of the chains and, working down the chains, liberate cellobiose and some glucose. Finally, β-glucosidases completes the saccharification by breaking cellobiose and small cello-oligosaccharides into glucose molecules [6].

Cellulases are inducible enzymes which are synthesized by microorganisms during their growth on cellulosic materials. Several microorganisms can produce cellulase enzyme including fungi, bacteria and actinomycetes. Presently, the majority of the commercial and laboratory cellulases are achieved by fungi due to their high enzyme activity, but several factors suggest that bacteria may have excellent potential [7]. Bacteria frequently have a higher growth rate than fungi allowing for higher rate of enzyme production. Most significantly, they show affinity to be more heat stable and are easier for genetic purpose. Various bacterial genera reported for cellulolytic activities include *Bacillus*, *Clostridium*, *cellulomonas*, *Rummminococcus*, *Alteromonas*, *Acetivibrio* etc. Among bacteria, *Bacillus* sp. including *B. brevis* [8], *B. pumilus* [9], *B. amyoliquefaciens* DL-3 [10], and *Bacillus subtilis* YJ1 [11] *Bacillus sp*. [12,13] are well recognized cellulase production under submerged condition [14,15]. The present study was to isolate a potential organic-solvent-thermostable alkalophilic cellulase producing bacteria from natural ecosystem. Afterthat, the cellulase enzyme was applied for purification and characterization of different parameters and purify enzyme was applied for the ethanol production and industrial exploitation which discuss in our next study.

## Results and discussion

### Isolation, screening and identification of thermotolerant cellulase producing bacterial cultures

Fifty (50) bacterial strains were isolated from the soil samples on CMC agar plates. For checking the cellulolytic activity of the isolates on plates, plates were stained with congo-red and NaOH solution. The zones of clearance by isolates reflect their extent of cellulolytic activity. Those having clearance zone greater than >1.0 cm were considered as significant. Among fifty (50) bacterial isolates, only twenty nine (29) bacterial isolates exhibited good cellulase activity which was reassessed by loading their culture broth in the wells on CMC agar plates which stained with congo-red and NaOH solution. The culture broth of good cellulase producers cleared more than >1.0 cm zone within 4–5 h of incubation at 65°C, thereby indicating an extra-cellular nature of the cellulase. The isolate RG-07, showing maximum clearance zone diameter was selected for further studies.

**Figure 1:**
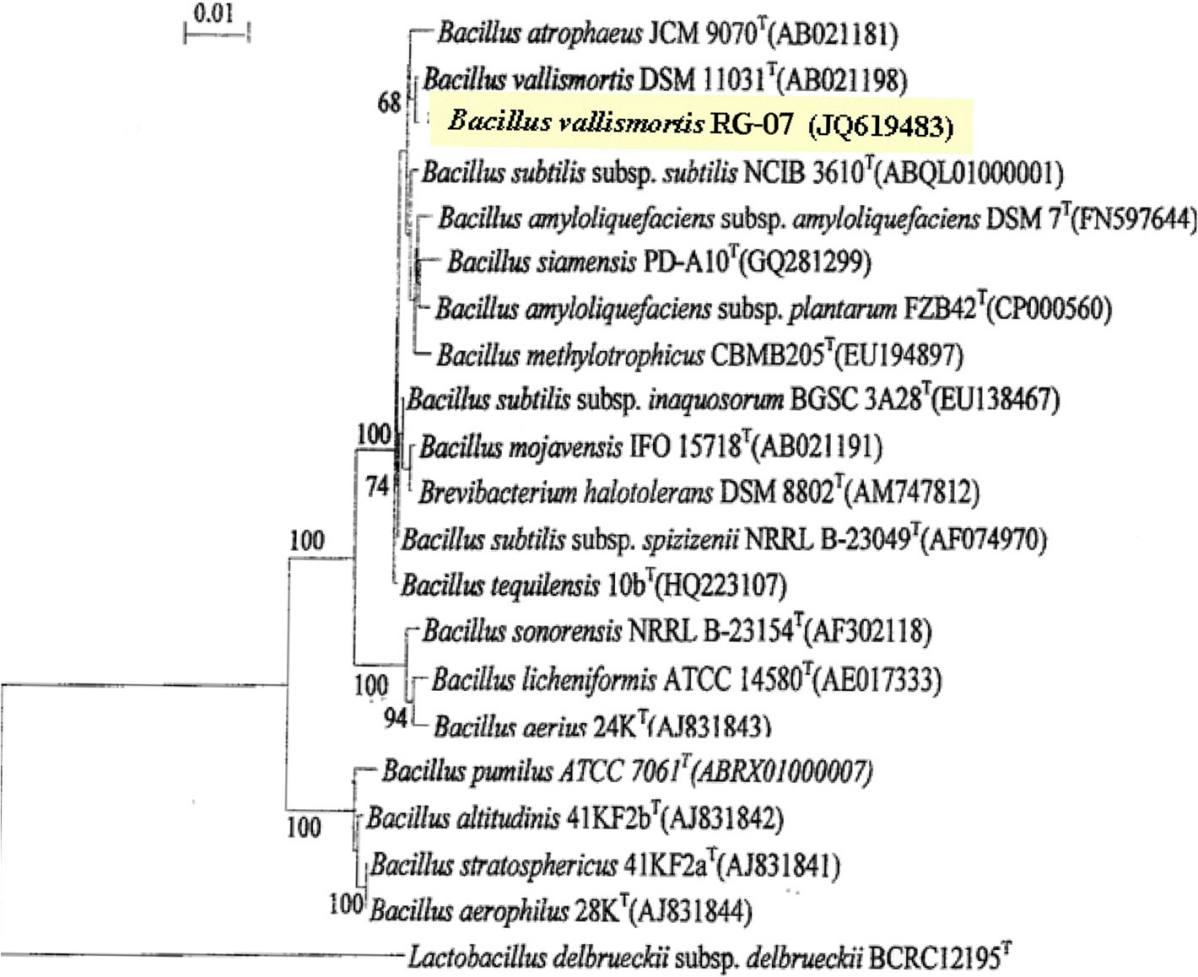
**Phylogenetic tree showing relation between Strain RG-07 and other** ***Bacillus*** **strains.** In phylogenetic tree analysis, the strain was in the same cluster with different strains of *Bacillus* and showed only 99.8% homology to other *Bacillus vallismortis* sp., so it could be stated that therefore it is different the from reported *Bacillus vallismortis*. The phylogenetic tree was drawn by MEGA 5 software using Neighbour-joining method and the significance of junctions was established using bootstrap method (1000 replicates).

The efficient strain RG-07 was rod-shaped, gram-positive, aerobe and facultative, motile, with positive acetylmethylcarbinol, catalase and oxidase test. It grew over a wide range of pH (4.0-11), temperature (10-85°C), NaCl concentration (0.0-12%), and was able to hydrolyze gelatin, casein, starch, tween-20, tween-40 and tween-80 and produce acid from glucose, xylose, mannitol and arabinose. It gives positive test for citrate utilization and nitrate reduction. The strain was halotolerant as it grew in the presence of 0.0-12% NaCl. On account of morphological and biochemical characteristics, it was identified as *Bacillus sp*. by MTCC IMTECH, Chandigarh (India). Analysis of 16S rDNA sequence revealed its 99.8% homology with *Bacillus vallismortis* strains, and was designated as *Bacillus vallismortis* RG-07. The 16S rDNA sequence was submitted to Gene bank [JQ: 619483] which doi is http://www.ncbi.nlm.nih.gov/nuccore/JQ619483 (Additional file 1). The strain RG-07 was in the same cluster of phylogenetic tree (Figure 1) with different strains of *Bacillus vallismortis.* However, the 16S rDNA sequence analysis indicates that it is a different and novel strain of *Bacillus vallismortis*.

### Effect of agro-waste materials on cellulase production

**Figure 2:**
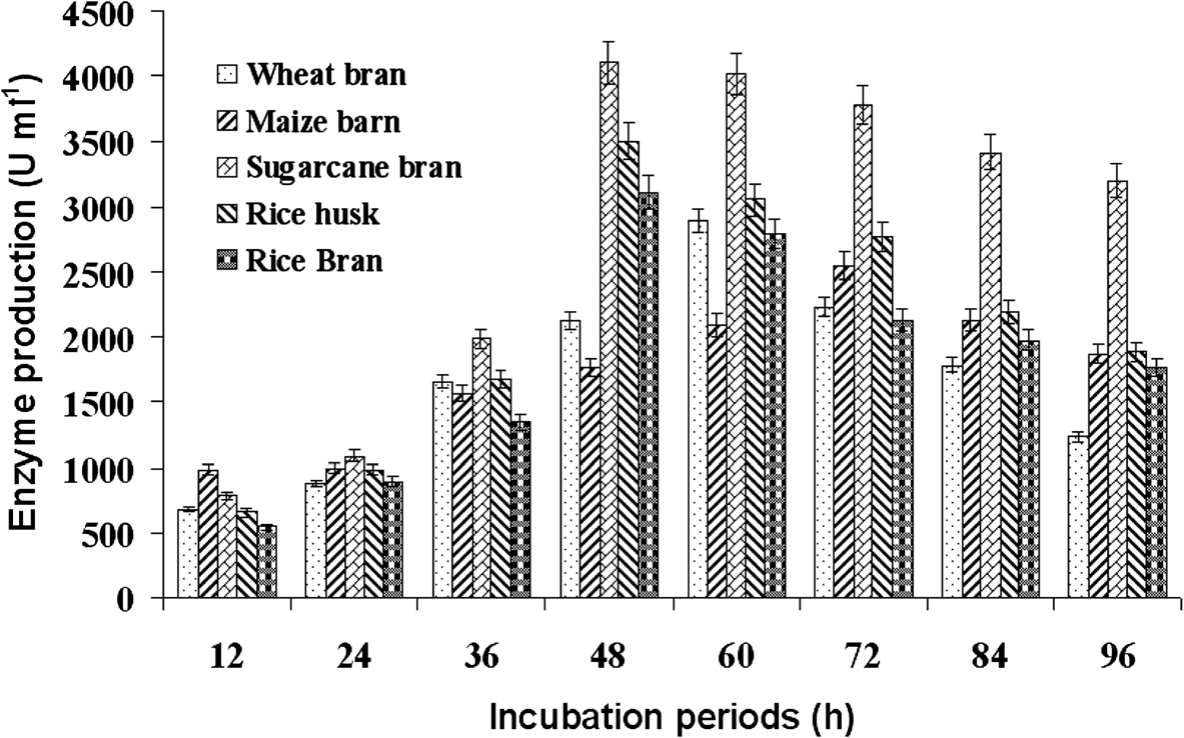
**Effect of different agro-waste materials on cellulase production by** ***Bacillus vallismortis*** **RG-07.** Five different agro-waste materials like sugarcane baggase, rice barn, rice husk, wheat bran and maize barn (2%) used as carbon sources for cellulose production, which inoculated with bacterial culture and incubated at 65°C, pH-7.0 for 12-96h.

The effect of agro-waste materials as carbon source on cell growth and cellulase production by *Bacillus vallismortis* was investigated with sugarcane baggase, rice bran, wheat bran, rice husk and maize bran. From Figure 2 it revealed that 2% sugarcane baggase (4105 U ml^−1^) was found as most suitable substrate for cellulase production followed by rice husk (3509 U ml^−1^) and rice bran (3110 U ml^−1^), within 48 h. Minimum cellulase production was reported from the wheat bran (2890 U ml^−1^) and maize bran (2545 U ml^−1^). Similarly, Sadhu et al. [16] also reported that sugarcane baggase was the best carbon sources for cellulase production by *Bacillus sp*. Annamalai et al. [17] and Jo et al. [14] were also reported that rice husk and rice bran were the best carbon source for cellulase production by *Bacillus halodurans* CAS 1 and *Bacillus amy-loliquefaciens* DL-3, respectively. The cellulosic waste materials such as rice bran, sugarcane baggase and rice hulls were used as best carbon sources for cellulase production due to their inducible nature [18]. The cellulase produced by the hydrolysis of cellulosic biomass by *Bacillus vallismortis* could be valuable for bio-ethanol production, single cell protein and other industrially required chemicals.

### Purification of extracellular cellulase

The crude enzyme extract was first concentrated by ammonium sulphate precipitation. Maximum activity was observed in the fraction obtained by the addition of ammonium sulphate in 80% with protein content of 21.54 mg ml^−1^. This fraction had 14864.94 U mg^−1^ of specific activity with recovery of 83.8% and with regard to purification it showed 4.7-fold purification (Table 1).

**Table 1:** **Purification of cellulase from** ***Bacillus vallismortis*** **RG-07**

**Purification steps**	**Total activity (U)**	**Total protein (mg)**	**Specific activity (U/mg)**	**Yield (%)**	**Purification fold**
Crude	3,81,788	120.75	3161.81	100	1.0
Ammonium sulphate	3,20,190.9	21.54	14864.94	83.8	4.7
Q-Sepharose	2,88089.4	7.79	36981.95	75.4	11.6
Sephadex G-75	109,968.9	0.89	123560.56	28.8	39.1

**Figure 3:**
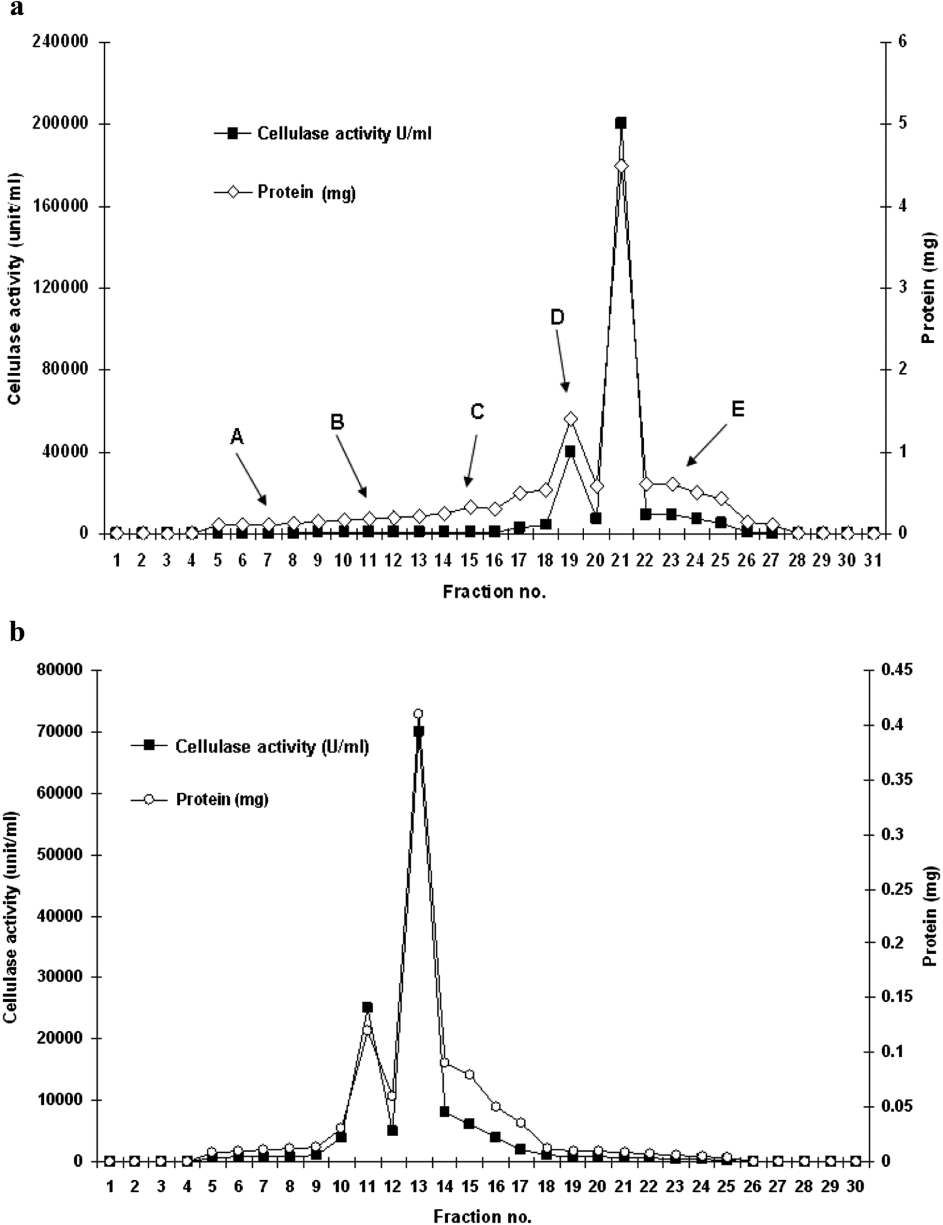
**Chromatographic purification profile of extracellular cellulase. a** Chromatographic purification profile of extracellular cellulase from *Bacillus vallismortis* RG-07 on anion-exchange column. Column was equilibrated with sodium phosphate buffer (100 mM, pH 7.0). The cellulase was eluted with a gradient of sodium chloride (0.1 M-0.5 M) in sodium phosphate buffer (100 mM, pH 7.0). A-0.1 M, B-0.2 M, C-0.3 M, D-0.4 M, E-0.5 M. **b** Chromatographic purification profile of extracellular cellulase on Sephadex G-75 gel filtration chromatography. Column was equilibrated with sodium phosphate buffer (100 mM, pH 7.0). The sample was loaded and eluted with the same buffer.

The active fraction of ammonium sulphate precipitation method was used for further purification by using ion exchange chromatography. Sample (1 ml) was loaded into the Q-Sepharose column pre-equilibrated with sodium phosphate buffer (100 mM, pH 7.0) and allowed to pass through the column. The un-bound fraction was collected and analyzed for cellulase activity and protein content. There was no cellulase activity in the un-bound fraction, while 1.8 mg ml^−1^ of protein was estimated. The absence of enzyme in un-bound fraction suggested that total cellulase was bound to matrix. The bound enzyme was eluted by sodium phosphate buffer (100 mM, pH 7.0) having NaCl with increasing concentration at gradient of 0.1 M. 10 ml solution of each concentration of NaCl was used to evade the bound enzyme. The cellulase activity was detected in the fraction released by the addition of 0.5 M NaCl anion-exchange chromatography of cellulase on column resulted in one prominent peak at the 21^th^ fraction (Figure 3a).

The active fraction was applied on Sephadex G-75 column. Figure 3b shows the fractionation pattern of cellulase on Sephadex G-75 column. One distinctive protein peak was appeared that overlapped with the cellulase activity. The purification process resulted in 39.1-fold purification factor and a final recovery of 28.8% of the enzyme with specific activity of 123560.56 U mg^−1^ (Table 1). Vijayaraghavan and Vincent, [12] reported 14.5-fold purified cellulase with 24% recovery after gel chromatography for purification of cellulase from *Bacillus* sp.

**Figure 4:**
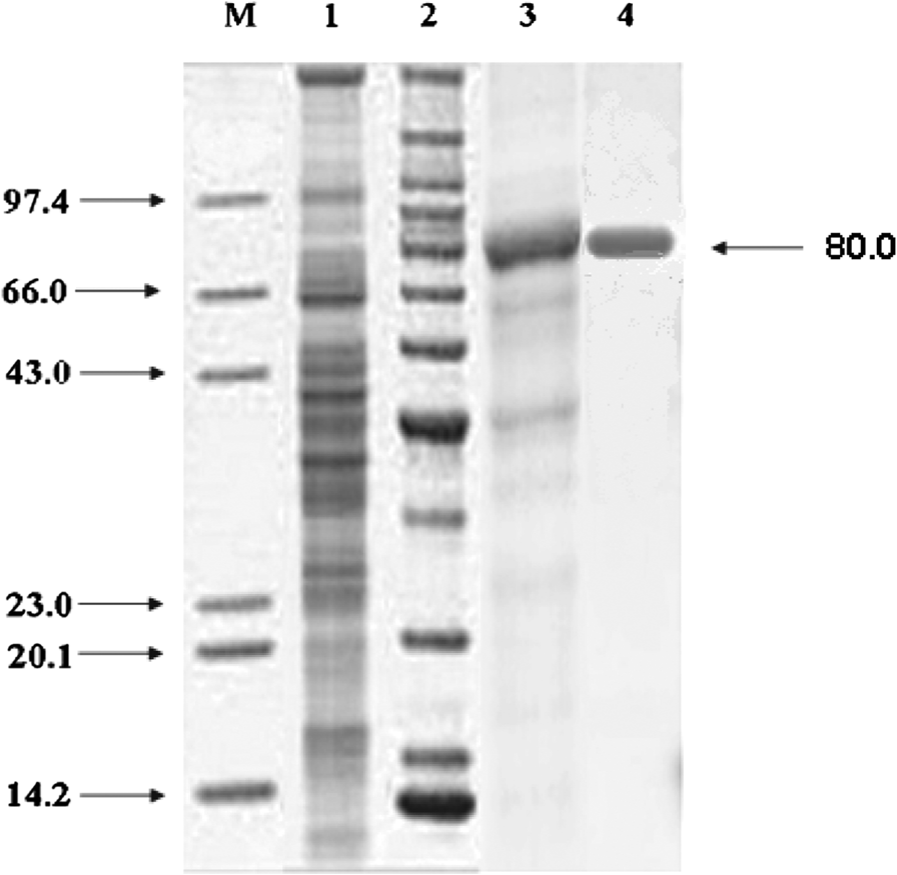
**SDS-PAGE of different fractions of cellulase of** ***Bacillus vallismortis*** **RG-07 obtained during purification steps.** Lane 1: Marker proteins; lane 2: Crude supernatant; lane 3: Concentrated enzyme after ammonium sulphate precipitation; lane 4: Purified enzyme obtained after anion-exchange chromatography; lane 5: Purified enzyme obtained after gel-filtration chromatography. Molecular weights were presented in the form of kDa.

The purity of the enzyme was confirmed by the presence of a single band on SDS-PAGE and its molecular weight was approximately 80 kDa (Figure 4), which was similar to alkalophilic *Bacillus* sp. HSH-810 where it is 80 kDa [19] but different from *Bacillus sp*. cellulase (54 kDa) [10,20].

### Characterization of purified enzyme

#### Effect of temperature on enzyme activity and stability

**Figure 5:**
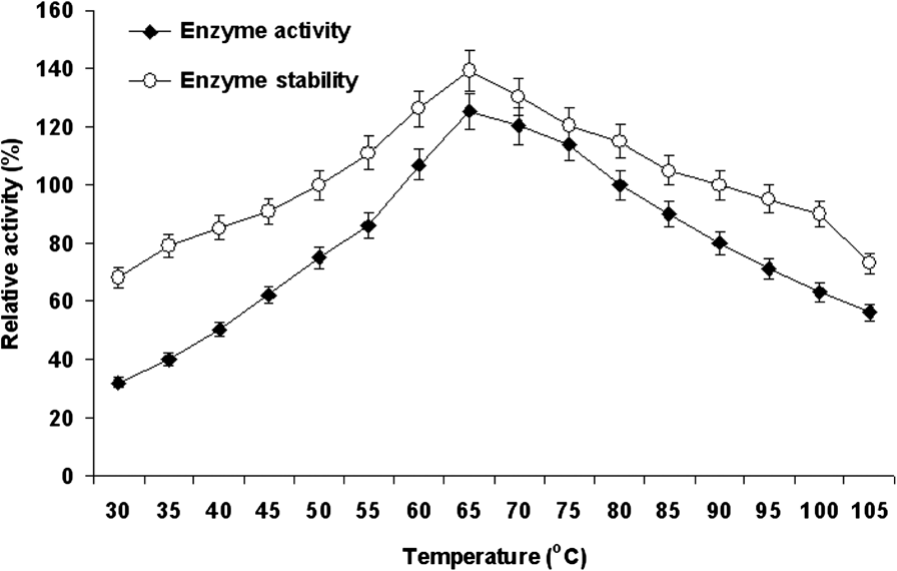
**Effect of temperature on enzyme activity and stability.** For enzyme activity reaction mixture was incubated at different temperatures (30-105°C) and for stability enzyme was pre-incubated at respective temperatures for 1h and reaction was conducted as standard assay method.

Effect of temperature on purified enzyme activity was recorded over a broad range of temperature (30-80°C) with the optimal activity at 65°C and declined thereafter (Figure 5). The cellulase of strain RG-07 was completely stable in the broad temperature range of 30-90°C during 1 h incubation. However, with further increase in every 5°C temperature, there was a gradual decrease in enzyme stability ranging between 15-20% upto 105°C. The enzyme retained 95, 90 and 73% activity even after treatment at 95, 100 and 105°C, respectively (Figure 5). Similarly, endoglucanase of *Bacillus licheniformis* C108 was highly stable up to 100°C [21]. The cellulase of strain RG-07 is more thermostable than cellulase studied by several other researchers. Most of workers have reported that thermostable cellulase stable up to 60-100°C but retained only ~50% activity at 100°C [22,21]. Most other thermotolerant *Bacillus* cellulase reported to so far, cellulases exhibited higher temperature optimum for activity and showed good thermal stability [23,24]. These are the properties considered to be very important for industrial cellulose saccharification. Hence it is evident that the cellulase of strain RG-07 is more thermostable, and may be applied to several biotechnological and industrial purposes.

#### Effect of pH on enzyme activity and stability

**Figure 6:**
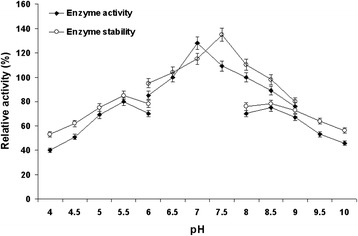
**Effect of pH on enzyme activity and stability.** For enzyme activity the reaction was assayed at respective pH and for stability enzyme was pre-incubated with buffers (100 mM, in ratio 1:1) of different pH (4–10) at 65°C for 1 h and assayed by standard assay method.

The pH activity and stability of the purified cellulase was determined by measuring the enzyme activity at varying pH values ranging from 4–10 using different suitable buffers. It observed that maximum cellulase activity was established at pH 7.0, however it was found to be most stable at pH 7.5 (Figure 6). Similar optimum enzyme activity was also found at pH 7.0 in *Pseudomonas fluorescence* [25], *Bacillus amyoliquefaciens* DL3 [10]. The relative activities at pH 4.0, 4.5, 5.0 5.5, 6.0, 6.5, 7.0, 7.5 and 8.0 were determined to be 53, 62, 75, 85, 95, 104, 135, 110 and 98%, respectively. At pH above 7.5, the cellulase activity decreased rapidly. The cellulase from *Bacillus vallismortis* RG-07 was stable in a range of pH 4.0-9.0 and at pH 10.0 approximately 80% of its activity was retained (Figure 6). Cellulases are generally stable over a wide range of pH from 5 to 10 [19,24].

#### Effect of metal ions on enzyme activity and stability

*Bacillus vallismortis* RG-07 cellulase was activated by 10 mM Ca^2+^, Mg^+2^, and Na^+1^ but inhibited by all other metal ions to a variable extent. Results suggest that cellulase showed maximum relative activity (184.4, 175.1 and 141.4%) and stability (160.3, 130.3, and 125.3%) in the presence of calcium, magnesium and sodium ions. Yoon et al. [26] and Bakare et al. [25] had also reported that Ca^2+^, Mg^+2^, and Na^+1^ ions strongly stimulated cellulase activity. Similarly Wang et al. [27] also reported that *Paenibacillus* sp. strain B39 showed maximum enzyme activity in the presence of 1 mM Ca^2+^. Cellulase activity was slightly inhibited was by Ni^2+^, Cu^2+^ and Zn^2+^ (Table 2). Cellulase was strongly inhibited in the presence of Mn^2+^, and Hg^2+^. Similar results were reported for *Bacillus* strain [23,16] and *Bacillus amyoliquefaciens* DL-3 [10]. It has been reported that the inhibition of cellulase activity by Hg^+^ ion might be related to its binding with thiol groups, tryptophan residue, or the carboxyl group of amino acid residues in the enzyme [28]. The inhibition of cellulase by Co^2+^, and Cu^2+^ ions could be due to competition between the exogenous cations and the protein-associated cations, resulting in decreased metallo-enzyme activity.

**Table 2:** **Effect of metal ions on enzyme activity and stability**

**Metal ions**	**Concentration (mM)**	**Residual activity (%)**	
		**Activity**	**Stability**
Control		100.0	100.0
CaCl_2_	5	156.4	125.4
	10	184.4	160.3
NiCl_2_	5	121.4	93.1
	10	90.4	87.7
FeSO_4_	5	112.9	100.8
	10	102	86.5
MgCl_2_	5	145.9	115.6
	10	175.1	130.3
CuSO_4_	5	87.4	82.5
	10	56.7	49.5
HgCl_2_	5	46	52
	10	25	32
MnCl_2_	5	76.9	83.9
	10	61.9	50.2
KCl	5	100.2	106.4
	10	92.1	90.1
NaCl	5	127.8	101.4
	10	141.4	125.3
ZnSO_4_	5	95	76
	10	63	59

Enzyme activity was determined at 65°C in the presence of metal ions in the reaction mixture directly and for stability enzyme was pre-incubated with different metal ions at 65°C for 1 h and assayed as standard assay method. The enzyme activity without incubation with metal ions was taken as 100%. Mean standard deviation for all the values is < ± 5.0%.

### Effect of organic solvents on cellulase stability

In another approach, the effect of various organic solvents (30%, v/v) on cellulase stability was also investigated for one week, and the results are depicted in Table 3. The cellulase of *Bacillus vallismortis* RG-07 is extraordinarily stable in the presence of all organic solvents under study. It was observed that except benzene, propanol and ethanol, presence of other solvents enhanced the cellulase activity. After incubation with n-dodecane, n-decane, xylene, iso-octane, toluene, n-haxane, n-butanol, acetone, methanol, and cyclohexane the cellulase activity increased to 242.5 (48 h), 178.7 (48 h), 117 (48 h), 170.3 (24 h), 180.2 (24 h), 143 (24 h), 160.3(24 h), 115.9 (24 h), 131.9 (48 h), and 133% (48 h), respectively. The presence of benzene, ethanol and propanol marginally reduced the cellulase with residual activities of 79.9, 88.2 and 84.2%, respectively (Table 3). Annamalai et al. [17] also reported an organic solvent stable alkaline cellulase of *Bacillus halodurans* CAS 1 strain rand with enhanced activity in the presence of organic solvents (25%, v/v). Zaks and Klibanov, [29] suggested that stimulation of enzyme activity by organic solvents might be due to the residues of carried-over non-polar hydrophobic solvent providing an interface, thereby keeping the enzyme in an open conformation which resulting stimulated activation. It is therefore; evident from our study that cellulase of *Bacillus vallismortis* RG-07 is remarkably stable in the presence of broad range hydrophilic as well as hydrophobic organic solvents employed in this study.

**Table 3:** **Stability of cellulase in presence of various organic solvents**

**Organic solvents (30%)**	**log** ***P***	**Residual activity (%)**							
		**1 h**	**24 h**	**48 h**	**72 h**	**96 h**	**120 h**	**144 h**	**168 h**
Methanol	−0.76	100.5	124.8	131.9	125.3	119.9	112.4	106.5	97.1
Iso-propanol	−0.28	90.7	104.3	99.5	92.5	91.8	91.0	88.3	84.2
Ethanol	−0.24	98	106.9	105.1	102.9	98.8	95.1	91.6	88.2
Benzene	2.13	99.7	112	120	109	96	89	85	79.9
Cyclohexane	3.3	90	118	133	123.9	112.9	109	93	89.9
Acetone	−0.23	105.2	115.9	110.9	104.1	101	96	92	90.3
Butanol	−0.80	120.7	160.3	145.2	130.4	122.6	112.3	107	100
Toluene	2.5	120.9	180.2	150.4	128.5	120.9	112.8	105.2	102.4
Iso-octane	2.9	120.5	170.3	135.6	130.2	123.2	120.4	115.6	100.2
Xylene	3.1	90	112	117	113	110	102	96	90
Hexane	3.6	128	143	124	128	113	114	104	100
n-decane	5.6	135.6	161.8	178.7	158.6	136.5	111.9	107	97.0
n-dodecane	6.0	141.6	170.7	242.5	168.4	179.8	130.5	113.0	100.0

Enzyme was pre-incubated with different organic solvents at a concentration of 30% (v/v) at 65°C for different time period and assayed as standard assay method. The enzyme activity without incubation with organic solvent was taken as 100%. Mean standard deviation for all the values is < ± 5.0%.

#### Effect of inhibitors on enzyme stability

When the *Bacillus vallismortis* RG-07 cellulase enzyme was incubated with EDTA, Iodo-acetic acid (IAA), *p*-chloromercuribenzoate (*p-*CMB), Dithiothreitol (DTT), Urea and β-mercaptoethanol, the enzyme activity was retained at 110%, 70%, 50%, 134%, 114%, and 167% of the original activity at 10 mM (Table 4). Similarly, Yin et al. [11] reported that *Bacillus subtilis* YJ1 retained full activity in the presence of 10 mM EDTA and β-mercaptoethanol, while Wang et al. [30] reported that cellulase of *Paenibacillus* sp. showed 22 and 29% activity with 10 mM. IAA and PCMB inhibits cellulase activity because they can bind with the SH group with different degree interaction and subsequently inhibit the activity. However, the β-mercaptoethanol and DTT can reduce the disulfide bonds and re-nature their activity, if the oxidation or aggregation of these enzyme proteins occurs during purification and storage. These phenomenons suggested that the active site of the enzyme contains -SH group [31].

**Table 4:** **Effect of different inhibitors on enzyme stability**

**Reagents**	**Concentration**	**Residual activity (%)**
Control		100.0
β–mercaptoethanol	0.1 (%)	179.6
	1.0 (%)	167.0
EDTA	5 mM	120.9
	10 mM	110.5
Urea	5 mM	119.0
	10 mM	114.0
IAA	5 mM	105.4
	10 mM	70.0
p-CMB	5 mM	90.0
	10 mM	50.0
DTT	5 mM	115.0
	10 mM	134.0

Enzyme was pre-incubated with different inhibitors at 65°C for 1 h and assayed as standard assay method. The enzyme activity without incubation with inhibitors was taken as 100%. Mean standard deviation for all the values is < ± 5.0%.

#### Effect of surfactant, detergent and oxidizing agents on enzyme stability

In order to have applications in detergent industries, cellulase must be stable to various detergent ingredients, such as surfactants. As shown in Table 5 the enzyme was appreciably stable in the presence of non-ionic surfactants like tween-40, tween-60, tween-80 and tritone-100 and detergent SDS. However, these compounds slightly inhibited the cellulase activity with 89.6, 93, 87, 92.5, 95.7% of residual activity at concentration 1.0% (v/v). Though, cellulase from a halotolerant isolate, *Bacillus* sp. L1 retained 91% activity towards triton X-100, tween-20, and tween-80 and lost about 41.1% activity in the presence of SDS [32]. Yin et al. [11] reported that, a highly thermo-stable and alkaline cellulase enzyme, there was 95% stability after 1 h incubation with SDS (10 mM). In another study, Sadhu et al. [16] and Wang et al. [30] have reported that SDS and tween-80 moderately inhibited cellulase activity 59-71% and 50-59%, respectively. Thus from the above study it clear that cellulase of *Bacillus vallismortis* RG-07 highly stable with 1% SDS (95%). This resistance, which is essential requirements, suggests that the enzyme may be used as an effective additive in detergents.

**Table 5:** **Effect of surfactants, commercial detergents and oxidizing agents on cellulase stability**

**Surfactants**	**Concentration (%)**	**Residual activity (%)**
Control		100.0
Tween-40	0.1	119.7
	1.0	89.6
Tween-60	0.1	124.9
	1.0	93.0
Tween-80	0.1	116.4
	1.0	87.0
Triton-X-100	0.1	105
	1.0	92.5
SDS	0.1	130
	1.0	95.7
Tide	0.1	90.7
	1.0	75.5
Ariel	0.1	99.7
	1.0	89.0
Surf Excel	0.1	94.3
	1.0	80
Fena	0.1	89.4
	1.0	68.4
Henko	0.1	88.2
	1.0	55.9
Sodium	0.1 (%)	87.9
perborate	0.5 (%)	68.2
	1.0 (%)	57.7
Sodium	0.1 (%)	127.2
hypochlorite	0.5 (%)	115.4
	1.0 (%)	94.6
H_2_O_2_	0.1 (%)	118.7
	1.0 (%)	96.9

Enzyme was pre-incubated with different surfactants, commercial detergents and oxidizing agents at 65°C for 1 h and assayed as standard assay method. The enzyme activity without incubation with surfactants, commercial detergents and oxidizing agents was taken as 100%. Mean standard deviation for all the values is < ± 5.0%.

The cellulase was substantially stable with commercial detergents at lower concentration (0.1%, w/v). However, higher concentration (1.0%, w/v) led to decrease the enzyme activity. The enzyme showed maximum stability in the presence of ariel, having residual activity of 89% after incubation at 65°C for 1 h (Table 5). The enzyme had 80%, 75.5, 68.4 and 55.9% residual activities in the presence of surf excel, tide, fena and henko, respectively under similar condition (Table 5). Similarly Annamalai et al. [17] reported that purified cellulase from *B. halodurans* CAS 1 retained its activity in the presence of some commercial detergents such as rin (85.33%), ariel (76.67%), henko (64.67%) and tide (80.33%). Sadhu et al. [16] also reported 57-72% residual activity with cellulase from *B*acillus strain in the presence of commercial detergents. Similar results are reported from *Thermonospora* sp. with different commercial detergent [33].

Among the oxidizing agents tested the cellulase activity enhanced in presence of sodium hypochlorite and H_2_O_2_ with residual activities 127.2 and 118.7% at concentration 0.1%, whereas, higher concentrations (0.5 and 1.0%) decreased the stability except sodium hypochlorite (115.4% residual activity at concentration 0.5%, w/v) (Table 5). Likewise, Wang et al. [27] also reported that lipase from *B. cepacia* was highly stable in the presence of hydrogen peroxide, sodium hypochlorite and sodium perborate after 1 h. The stability profile of the cellulase in the presence of detergents and oxidizing agents prove its potential application in the detergent formulations as these agents are the active components of house hold detergents.

### Kinetic analysis

**Figure 7:**
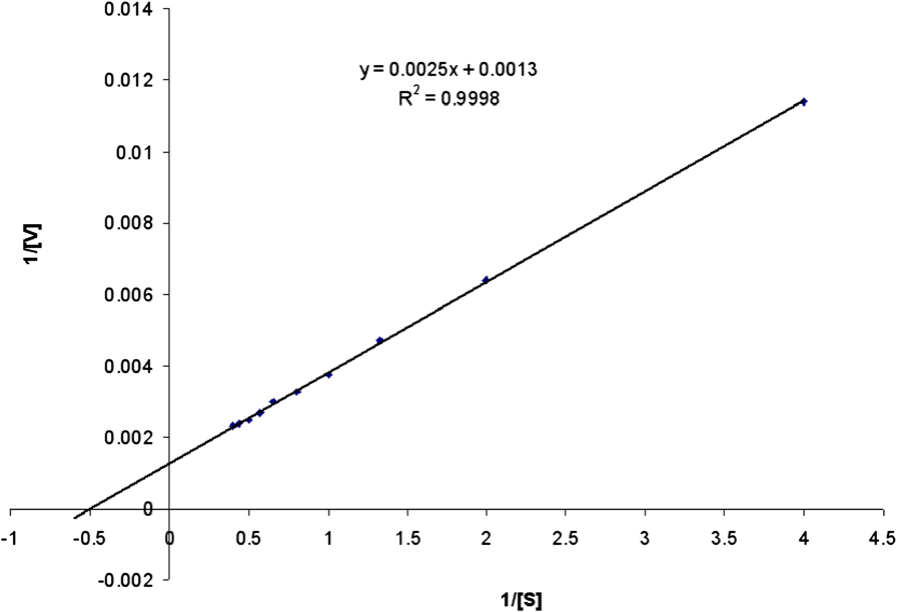
**Lineweaver-Burke plot of initial velocity data of** ***Bacillus vallismortis*** **RG-07 cellulase on CMC.** CMC concentration is 0.25, 0.5, 0.75, 1.0, 1.25, 1.5, 1.75, 2.0, 2.25, 2.5, 2.75, 3.0, 3.25, 3.5, 3.75, 4.0 mg ml^−1^, respectively.

Kinetic analysis with CMC revealed the K_m_ and V_max_ to be 1.923 mg ml^−1^ and 769.230 μg ml^−1^ min^−1^, respectively by Linweaver-Burk plot (Figure 7). Mawadza et al. [23] also reported similar value of K_m_ (1.5 and 1.7 mg ml^−1^) and V_max_ (1.5 and 0.9 mmol min^−1^ mg^−1^) from *Bacillus* sp. using CMC as substrate. If the K_m_ value is low, it means that enzyme had a stronger affinity with substrate.

## Conclusion

It is the first instance when a thermo-tolerant cellulase being reported from a thermo-tolerant solvent tolerant *Bacillus vallismortis* RG-07 isolate. The strain is unique with respect to increased cellulase activity in the presence of various solvents of greater hydrophobicity (log P ≥ 4.5). The cellulase activity in broad pH and temperature range of 4.0-10.0 and 30-105°C clearly indicate the thermo-alkaline nature of this enzyme. Considering its stability under high temperature as well as mild alkaline condition, cellulase may be exploited for industrial usage.

## Methods

### Isolation, screening and identification of thermo-tolerant cellulase producing bacteria

The soil samples were collected aseptically from different garden site of University campus (Dr. Ram Manohar Lohia Avadh University Faizabad) to isolate cellulase producing bacteria. 1 g soil was suspended in 9.0 ml sterile distilled water, agitated for a min and 0.1 ml suspension was spread over CMC agar plates (pH 7.0) containing, 2.0%, CMC; 0.5%, Ammoium sulphate; 2%, agar and incubated at 65°C, till sufficient growth appeared. After sufficient growth incubated plates were overlaid with congo-red solution (0.1%) for 10 min and then washed with 1 N NaOH solution for de-staining. If a strain was cellulolytic then it started hydrolyzing the cellulose present in the surrounding and in the zone degradation there was no red color formation. Selection was done as per colonies with and without clear and transparent zone as cellulase producing and cellulase non-producing strain, respectively. Bacterial colonies showing clear zones were selected, streaked twice on CMC agar plates for purification and maintained as pure culture over CMC agar slants (pH 7.0, 4°C). The isolate having maximum clearance zone was selected for further studies. The selected bacterial isolate RG-07 was identified by morphological and biochemical characterization as per the Bergey’s Manual of Systematic Bacteriology [34]. The identity of RG-07 was authenticated from Institute of Microbial Technology (IMTECH), Chandigarh, India based on the phenotypic (16S r DNA) and biochemical tests.

### Crude enzyme preparation and enzyme assay

To obtain crude enzyme 24 h old cultures were transferred to micro-centrifuge tubes and centrifuged at 12000 rpm for 10 min. Cells were discarded and resultant supernatant was used as the crude enzyme for various enzyme assay. Cellulase was assayed by measuring the reducing sugar released by reaction on CMC. Cellulase assay was done by Nelsom [35] and Somogy [36] methods, in this method a reaction was performed in which a reaction mixture contain 500 μl of substrate (1.0% soluble CMC in 1.0 M phosphate buffer pH 7.0.), 100 μl of the enzyme and 400 μl distilled water. The reaction mixture was incubated for 10 min at 65°C. Reaction was stopped by adding 1 ml of alkaline copper tartrate solution and incubated in boiling water bath for 10 min and cooled, then added arsenomolybdate solution for color stabilization. Optical density of each sample with reaction mixture was taken at 620 nm in a spectrophotometer (Shimadzu, Japan). One unit of enzyme activity was defined as the amount of enzyme that liberates 1.0 μg of glucose min/ml.

### Effect of agro-waste materials on cellulase production

To study the effect of agro-waste materials for cellulase production, growth was carried out in 150 ml Erlenmeyer flasks in 50 ml of basal medium contained; 0.05% ammonium sulphate, 0.5% MgSO_4_· 7H_2_O (pH 7.0) supplemented with 2% (w/v) agro-waste materials to be investigated such as sugarcane baggase, rice bran, rice husk, wheat bran, and maize bran at 65°C for 12–96 h. The enzyme activity was estimated by Nelsom [35] and Somogy [36] methods as discuss in enzyme essay section.

### Cellulase purification

The crude culture supernatant obtained from 24 h old cultures of *Bacillus vallismortis* RG-07 grown under optimal conditions was subjected to purification. All the purification steps were carried out at 4°C. The crude culture filtrate was subjected to a cooling centrifugation at 10,000 rpm to remove the cells and the residual medium. Supernatant was precipitated overnight with (NH_4_)_2_SO_4_ (80% saturation) and the pellets was recovered by centrifugation at 12000 rpm for 10 min. The pellet was re-suspended in a small amount 100 mM phosphate buffer, pH 7.0, and dialyzed overnight against the same buffer. The corresponding precipitates were recovered, dissolved individually in fresh buffer and assayed for both total protein content and cellulase activity. The dialyzed sample was applied to a Q-Sepharose (Sigma-Aldrich, USA) column equilibrated with sodium phosphate buffer (100 mM, pH 7.0). The desired enzyme fraction was allowed to bind with matrix for 2 h at 4°C. The unbound fraction was collected and analyzed for enzyme activity and for protein content. The bound fractions were eluted with a linear gradient of NaCl (0.1-0.5 M, 10 ml each) in the same buffer at a flow rate of 1.0 ml min^−1^.

The active fractions were collected and dialyzed against sodium phosphate buffer (pH 7.0). The dialyzed sample was further purified by gel-filtration chromatography for purification up to homogeneity. The Sephadex-75 column (Sigma Aldrich Pvt. Ltd., USA, 1.5 × 40 cm) was equilibrated with sodium phosphate buffer (100 mM, pH 7.0) and 1 ml of concentrated sample was applied to the column. The flow rate was adjusted to 5–6 ml h^−1^ and fraction of 2 ml each was collected. Cellulase activity and estimation of protein content were determined for each individual fraction.

### Determination of protein concentrations

The protein content of individual fraction obtained after different steps of chromatography was monitored by measuring the extinction at 280 nm. Quantified protein for each fraction was done by the method of Lowry et al. [37] using Bovine serum albumin (BSA) as standard and expressed as mg ml^−1^.

### Polyacrylamide gel electrophoresis

The active fraction, with maximum specific activity, obtained after gel filtration chromatography along with crude, ammonium sulphate and anion-exchange chromatography was electrophorezed by Sodium Dodecyl Sulphate-Poly Acrylamide Gel Electrophoresis in a 12.5% polyacrylamide gel according to the method of Laemmli [38]. Approximate molecular weight of the cellulase was estimated by SDS-PAGE against the molecular mass markers i.e. lysozyme (14.3 kDa), β-lactoglobulin (20 kDa), Carbonic anhydrase (29 kDa), ovalbumin (43 kDa), bovine serum albumin (66 kDa) and phosphorylase B (97.4 kDa) (Sigma-Aldrich Pvt Ltd., USA) run with the samples.

### Characterization of purified enzyme

#### Effect of temperature on enzyme activity and stability

The influence of temperature on activity of cellulase was studied by incubating the reaction mixture at different temperatures (30, 35, 40, 45, 50, 55, 60, 65, 70, 75, 80, 85, 90, 95, 100, and 105°C). The enzyme was incubated at different temperatures (30, 35, 40, 45, 50, 55, 60, 65, 70, 75, 80, 85, 90, 95, 100, and 105°C) for 1 h to study the stability of the enzyme. The residual cellulase activity was measured by conducting the reaction at temperature 65°C and pH 7.0 [35,36]. The activity of the enzyme was considered as 100% under standard assay conditions.

#### Effect of pH on cellulase activity and stability

The effect of pH on cellulase activity was measured in the pH range of 4 to 10, using the appropriate buffers at concentration of 100 mM (4.0-6.0, sodium acetate; 6.0-8.0, sodium phosphate; 8.0-10.0, Tris–HCl) under standard assay conditions [35,36]. To study stability as a function of pH, 100 μl of the purified enzyme was mixed with 100 μl of the buffer solutions and incubated at 65°C for 1 h then aliquots of the mixture were taken to measure the residual cellulase activity (%) under standard assay conditions [35,36].

#### Effect of metal ions on enzyme activity and stability

The effect of various metal ions (5 mM and 10 mM) on enzyme activity was investigated using FeSO_4,_ CaCl_2,_ KCl, NaCl, MgCl_2,_ MnCl_2,_ ZnSO_4,_ CuSO_4,_ HgCl_2_ and NiCl_2._ The enzyme was incubated with different metals at 65°C for 1 h to study metal ion stability and assayed under standard assay conditions [35,36].

#### Effect of organic solvent on cellulase stability

Purified enzyme having maximum cellulase activity was incubated with 30% (v/v) of different organic solvent viz., n-dodecane, n-decane, iso-octane, xylene, n-hexane, n-butanol, cyclohexane, acetone, toluene, benzene, ethanol, methanol and propanol for one week in screw crapped tubes at 65°C and 120 rpm. The residual cellulase activity was estimated against the control, in which solvent was not present [35,36].

#### Effect of inhibitors on cellulase activity

The effects ethylene diamine tetra acetic acid (EDTA), *β*-mercaptoethanol, Phenyl methyl sulphonyl flouride (PMSF) and urea as inhibitors on cellulase activity were investigated at a concentration of 5 mM and 10 mM in order to characterize enzyme. Crude enzyme was pre-incubated with the above mentioned reagents for 1 h at 65°C and residual activity (%) was determined under standard assay conditions [35,36].

#### Effect of surfactants, commercial detergents and oxidizing agents on enzyme stability

The cellulase sample was incubated with surfactants *viz*., Triton-X-100, Tween-40, Tween-60, Tween-80, SDS (0.1 and 1.0%, v/v), commercial detergents *viz*., surf, aerial, ghari, henko and fena (0.1 and 1.0%, w/v), and oxidizing agents *viz*., H_2_O_2_ (0.1 and 1.0%, v/v)_,_ sodium perborate and sodium hypochlorite (0.1, 0.5 and 1.0%, v/v) for 1 h at 65°C and then the residual activity (%) was tested under standard assay conditions [35,36].

### Kinetic analysis

The influence of substrate concentration on the reaction velocity of the purified cellulase was studied with CMC [39]. The purified cellulase was incubated with various concentration of CMC ranged from 0.25-4.0 mg ml^−1^. In all cases, the enzymatic activity was assayed under standard conditions. The Michaelis constant (K_m_) and maximum velocity (V_max_) was determined from Lineweaver-Burk plots of Michaelis-Menten equation [40].

### Statistical analysis

Each experiment was performed twice, each in triplicate and standard deviation for each experimental results were calculated using the Microsoft Excel.

### Availability of Supporting Data’ (ASD)

Name of the repository is NCBI (National Center for Biotechnology Information) where our data is deposited and a link to the dataset DOI is http://www.ncbi.nlm.nih.gov/nuccore/JQ619483. The supporting data also include in this manuscript as a supporting file [41].

## Additional file

Additional file 1:
**Bacillus vallismortis strain RG-07 16S ribosomal RNA gene, partial sequence.**
