# Supplementary material for: Isolation, production, purification and characterization of an organic-solvent-thermostable alkalophilic cellulase from Bacillus vallismortis RG-07
Source: BMC Biotechnol. 2015 Mar 19;15:19. doi: 10.1186/s12896-015-0129-9 (PMC4377051; doi:10.1186/s12896-015-0129-9)
Supplement: Additional file 1: — Bacillus vallismortis strain RG-07 16S ribosomal RNA gene, partial sequence. [file 12896_2015_129_MOESM1_ESM.doc]

# Bacillus vallismortis strain RG-07 16S ribosomal RNA gene, partial sequence

GenBank: JQ619483.1

[FASTA](http://www.ncbi.nlm.nih.gov/nuccore/386778762?report=fasta) [Graphics](http://www.ncbi.nlm.nih.gov/nuccore/386778762?report=graph)

- [Features](http://www.ncbi.nlm.nih.gov/nuccore/JQ619483#feature_386778762)
- [Sequence](http://www.ncbi.nlm.nih.gov/nuccore/JQ619483#sequence_386778762)

LOCUS JQ619483 1396 bp DNA linear BCT 13-MAY-2012

DEFINITION Bacillus vallismortis strain RG-07 16S ribosomal RNA gene, partial

sequence.

ACCESSION JQ619483

VERSION JQ619483.1 GI:386778762

KEYWORDS .

SOURCE Bacillus vallismortis

ORGANISM [Bacillus vallismortis](http://www.ncbi.nlm.nih.gov/Taxonomy/Browser/wwwtax.cgi?id=72361)

Bacteria; Firmicutes; Bacilli; Bacillales; Bacillaceae; Bacillus.

REFERENCE 1 (bases 1 to 1396)

AUTHORS Rai,P., Tiwari,S. and Gaur,R.

TITLE Direct Submission

JOURNAL Submitted (21-JAN-2012) Department of Microbiology, Dr. Ram Manohar

Lohia Avadh University, Allahabad Road, Faizabad, Uttar Pradesh

224001, India

FEATURES Location/Qualifiers

source 1..1396

/organism="Bacillus vallismortis"

/mol_type="genomic DNA"

/strain="RG-07"

/isolation_source="soil"

/db_xref="taxon:[72361](http://www.ncbi.nlm.nih.gov/Taxonomy/Browser/wwwtax.cgi?id=72361)"

/country="India: Faizabad, Uttar Pradesh"

/PCR_primers="fwd_name: 27f, fwd_seq:

agagtttgatcctggctcag, rev_name: 1492r, rev_seq:

tacggttaccttgttacgactt"

[rRNA](http://www.ncbi.nlm.nih.gov/nuccore/386778762?from=1&to=1396&sat=4&sat_key=69146704) <1..>1396

/product="16S ribosomal RNA"

ORIGIN

1 tcctggctca ggacgaacgc tggcggcgtg cctaatacat gcaagtcgag cggacagatg

61 ggagcttcct ccctgatgtt agcggcggac gggtgagtaa cacgtgggta acctgcctgt

121 aagactggga taactccggg aaaccggggc taataccgga tgcttgtttg aaccgcatgg

181 ttcaaacata aaaggtggct tcggctacca cttacagatg gacccgcggc gcattagcta

241 gttggtgagg taatggctca ccaaggcaac gatgcgtagc cgacctgaga gggtgatcgg

301 ccacactggg actgagacac ggcccagact cctacgggag gcagcagtag ggaatcttcc

361 gcaatggacg aaagtctgac ggagcaacgc cgcgtgagtg atgaaggttt tcggatcgta

421 aagctctgtt gttagggaag aacaagtgcc gttcaaatag ggcggcacct tgacggtacc

481 taaccagaaa gccacggcta actacgtgcc agcagccgcg gtaatacgta ggtggcaagc

541 gttgtccgga attattgggc gtaaagggct cgcaggcggt ttcttaagtc tgatgtgaaa

601 gcccccggct caaccgggga gggtcattgg aaactgggga acttgagtgc agaagaggag

661 agtggaattc cacgtgtagc ggtgaaatgc gtagagatgt ggaggaacac cagtggcgaa

721 ggcgactctc tggtctgtaa ctgacgctga ggagcgaaag cgtggggagc gaacaggatt

781 agataccctg gtagtccacg ccgtaaacga tgagtgctaa gtgttagggg gtttccgccc

841 cttagtgctg cagctaacgc attaagcact ccgcctgggg agtacggtcg caagactgaa

901 actcaaagga attgacgggg gcccgcacaa gcggtggagc atgtggttta attcgaagca

961 acgcgaagaa ccttaccagg tcttgacatc ctctgacaat cctagagata ggacgtcccc

1021 ttcgggggca gagtgacagg tggtgcatgg ttgtcgtcag ctcgtgtcgt gagatgttgg

1081 gttaagtccc gcaacgagcg caacccttga tcttagttgc cagcattcag ttgggcactc

1141 taaggtgact gccggtgaca aaccggagga aggtggggat gacgtcaaat catcatgccc

1201 cttatgacct gggctacaca cgtgctacaa tggacagaac aaagggcagc gaaaccgcga

1261 ggttaagcca atcccacaaa tctgttctca gttcggatcg cagtctgcaa ctcgactgcg

1321 tgaagctgga atcgctagta atcgcggatc agcatgccgc ggtgaatacg ttcccgggcc

1381 ttgtacacac cgcccg
